# Three-dimensional architecture of the whole human soleus muscle *in vivo*

**DOI:** 10.7717/peerj.4610

**Published:** 2018-04-18

**Authors:** Bart Bolsterlee, Taija Finni, Arkiev D’Souza, Junya Eguchi, Elizabeth C. Clarke, Robert D. Herbert

**Affiliations:** 1 Neuroscience Research Australia (NeuRA), Sydney, New South Wales, Australia; 2 University of New South Wales, Sydney, New South Wales, Australia; 3 Faculty of Sport and Health Sciences, University of Jyväskylä, Jyväskylä, Finland; 4 Murray Maxwell Biomechanics Laboratory, Institute for Bone and Joint Research, Kolling Institute of Medical Research, Sydney Medical School, University of Sydney, Sydney, New South Wales, Australia

**Keywords:** Soleus, Muscle architecture, Passive muscle properties, Diffusion tensor imaging, MRI

## Abstract

**Background:**

Most data on the architecture of the human soleus muscle have been obtained from cadaveric dissection or two-dimensional ultrasound imaging. We present the first comprehensive, quantitative study on the three-dimensional anatomy of the human soleus muscle *in vivo* using diffusion tensor imaging (DTI) techniques.

**Methods:**

We report three-dimensional fascicle lengths, pennation angles, fascicle curvatures, physiological cross-sectional areas and volumes in four compartments of the soleus at ankle joint angles of 69 ± 12° (plantarflexion, short muscle length; average ± SD across subjects) and 108 ± 7° (dorsiflexion, long muscle length) of six healthy young adults. Microdissection and three-dimensional digitisation on two cadaveric muscles corroborated the compartmentalised structure of the soleus, and confirmed the validity of DTI-based muscle fascicle reconstructions.

**Results:**

The posterior compartments of the soleus comprised 80 ± 5% of the total muscle volume (356 ± 58 cm^3^). At the short muscle length, the average fascicle length, pennation angle and curvature was 37 ± 8 mm, 31 ± 3° and 17 ± 4 /m, respectively. We did not find differences in fascicle lengths between compartments. However, pennation angles were on average 12° larger (*p* < 0.01) in the posterior compartments than in the anterior compartments. For every centimetre that the muscle-tendon unit lengthened, fascicle lengths increased by 3.7 ± 0.8 mm, pennation angles decreased by −3.2 ± 0.9° and curvatures decreased by −2.7 ± 0.8 /m. Fascicles in the posterior compartments rotated almost twice as much as in the anterior compartments during passive lengthening.

**Discussion:**

The homogeneity in fascicle lengths and inhomogeneity in pennation angles of the soleus may indicate a functionally different role for the anterior and posterior compartments. The data and techniques presented here demonstrate how DTI can be used to obtain detailed, quantitative measurements of the anatomy of complex skeletal muscles in living humans.

## Introduction

The macroscopic arrangement of muscle fibres in the muscle belly is referred to as muscle architecture. Muscle architecture is often quantified by parameters such as fascicle length, pennation angle and physiological cross-sectional area (PCSA). Muscle architecture differs markedly between muscles and individuals (Ward et al., 2009; Woittiez, 1984), and changes with age (Narici, Maffulli & Maganaris, 2008; Siebert et al., 2017; Weide et al., 2015), exercise (Blazevich, 2006) and disease (Shortland et al., 2002; Foran et al., 2005). To study these processes, quantitative methods to measure muscle- and subject-specific architectural parameters are required. In this study, we use magnetic resonance imaging (MRI) techniques to quantify the architecture of the human soleus muscle *in vivo*.

The human soleus has a complex, three-dimensional (3D) architecture. Studies of cadaver muscles using micro-dissection techniques and magnetic resonance imaging have shown that the soleus is compartmentalised: it consists of a unipennate posterior part wrapped around a radially bipennate anterior part (Agur et al., 2003; Hodgson et al., 2006). The fascicles in each compartment have distinctly different orientations but similar lengths (Agur et al., 2003). The large volume (∼425 cm^3^ *in vivo* (Lee et al., 2006)) and short fascicle lengths (3–4 cm (Agur et al., 2003)) give the soleus the largest physiological cross-sectional area of any human lower limb muscle (Ward et al., 2009).

The complexity of the soleus’ architecture is also reflected in the connective tissues, examined in great detail by Hodgson et al. (2006). Distally, the muscle is connected to the calcaneus through the rope-like Achilles tendon, which the soleus shares with the gastrocnemius muscle. Just proximal to its insertion on the calcaneus, the Achilles tendon has an elliptical cross-section in the transverse plane, but more proximally it becomes wider and thinner as it joins the sheet-like posterior aponeurosis of the soleus (Hodgson et al., 2006; Finni et al., 2003b; Balius et al., 2013). The distal ends of muscle fascicles in the posterior compartment of the soleus insert on the posterior aponeurosis and the proximal ends of the muscle fascicles originate from the posterior side of the anterior aponeurosis. The anterior aponeurosis (the origin of the muscle) forms another curved sheet of connective tissue that extends almost the entire length of the muscle belly, connecting the muscle proximally to the tibia and fibula and separating the muscle into posterior and anterior compartments. The anterior aspect of the anterior aponeurosis provides the origin of muscle fascicles in the anterior compartment. Fascicles in the anterior compartment insert on a protrusion of the posterior aponeurosis called the medial septum, which separates the muscle into medial and lateral compartments and presents as a clearly identifiable T-shaped structure on transverse MRI images (Hodgson et al., 2006). The structural partitioning of the human soleus is also evident in the 3D branching of nerves (Loh, Agur & McKee, 2003), indicating that compartments may have functionally different roles (English, Wolf & Segal, 1993).

There have been few reports of quantitative measurements of the three-dimensional architecture of the soleus *in vivo*. Quantification of the 3D architecture of the soleus *in vivo* is difficult using conventional techniques such as ultrasound. With ultrasound, measurements from the deeper (anterior and proximal) compartment of the soleus muscle is difficult because image quality is often poor, although this depends on the system (Barber et al., 2017; Rana, Hamarneh & Wakeling, 2013; Lai et al., 2015; Chow et al., 2000; Martin et al., 2001). Also, in the soleus it is difficult to orient the transducer in the plane of fascicles and perpendicular to the aponeurosis (Rana, Hamarneh & Wakeling, 2013), as is required for accurate measurements of muscle architecture (Bolsterlee, Gandevia & Herbert, 2016; Lee et al., 2015).

Three-dimensional imaging techniques such as magnetic resonance imaging (MRI) overcome some of the limitations of ultrasound (Finni et al., 2003a; Balius et al., 2013; Hodgson et al., 2006). MRI has been used to quantify lengths of the whole muscle belly and the structure of connective tissues, and to map intramuscular velocities during isometric contractions (Finni et al., 2003a; Hodgson et al., 2006). However, anatomical MRI scans lack the resolution to discern individual muscle fibres, precluding measurement of key indices of muscle architecture such as fascicle lengths and pennation angles. For this reason, there are limited data on fascicle lengths and pennation angles in the human soleus *in vivo* (see Agur et al. (2003) for an overview of the available data). Also, it is largely unknown whether architectural parameters are uniform or differ between compartments. Non-uniformities could indicate as yet unrevealed functional differences between the soleus compartments.

Here, we use diffusion tensor imaging (DTI) to quantify the three-dimensional architecture of the human soleus muscle *in vivo* (Damon et al., 2017; Van Donkelaar et al., 1999; Bolsterlee et al., 2017; Oudeman et al., 2015). DTI is a magnetic resonance imaging (MRI) technique which exploits the principle that longitudinally arranged microstructures in muscles (such as cell membranes) hinder the diffusion of water molecules more in the plane perpendicular to the muscle fibre’s long axis than along that axis. This principle can be used to measure fibre orientation (Damon et al., 2002) and—most interestingly for anatomical studies—to quantify the 3D muscle architecture using DTI fibre tractography algorithms (Damon et al., 2017; Yeh et al., 2013; Mori et al., 1999).

We have recently extended DTI tractography techniques by constraining the fibre tracts to terminate at the surface of the muscle (Bolsterlee et al., 2017). The muscle surface is located using MRI. Here, we apply these novel techniques that combine anatomical MRI with DTI data to quantify the complex architecture of the human soleus *in vivo* at two different muscle lengths. We provide the most detailed data to date of soleus muscle architecture, and changes in soleus muscle architecture with passive lengthening.

## Materials & Methods

We further analysed MRI and DTI data obtained from the left lower legs of six subjects (Table 1) of a previous study of the architecture of the medial gastrocnemius muscle (Bolsterlee et al., 2017). All procedures conformed to the Declaration of Helsinki (2008) and were approved by University of New South Wales’ Human Research Ethics Committee (HC15006; *in vivo* data) or Northern Sydney Local Health District (RESP/16/9; cadaver data). Written informed consent of all subjects was obtained prior to their participation.

**Table 1:** Characteristics of participants and joint positions.

Characteristic	Value
Age (years)	29.3 ± 5.2
Gender (M:F)	3:3
Height (cm)	168.0 ± 6.8
Weight (kg)	62.9 ± 4.1
Shank length (cm)^a^	39.3 ± 2.2
Knee angle (°)	20 ± 5
Ankle angle in ‘short’ position (°)^b^	69 ± 12
Ankle angle in ‘long’ position (°)^b^	108 ± 7
Change in muscle–tendon length (mm)	30.0 ± 8.0

**Notes.**

a Shank length was measured as the distance from the lateral femoral condyle to the middle of the lateral malleolus.

b Ankle joint angle is the angle between the tibia and the sole of the foot, where 90° is a neutral ankle joint orientation and values below and above 90° indicate plantarflexion and dorsiflexion, respectively.

Of the original eight participants in our previous study (Bolsterlee et al., 2017), data from one participant were excluded because of poor image quality, thought to have been caused by movement during scanning. Data from another participant were excluded because DTI fibre tractography generated sparse fibre tracts (see section “Muscle architecture measurements”).

### MRI and DTI scans

For each participant, we analysed two sets of MRI and DTI scans obtained under passive conditions with the ankle in a dorsiflexed position (condition ‘long’) and in a plantarflexed position (condition ‘short’). An MRI-compatible frame was used to maintain the ankle in a fixed position. The frame could be locked at different angles to reposition the ankle. For all scans, the participants lay supine with the knee slightly flexed (Table 1). The ankle was passively dorsiflexed to an angle that corresponded to the slack length of the medial gastrocnemius muscle, determined using ultrasound imaging (mean ± SD = 69 ± 12°, where 90° means the sole of the foot is perpendicular to the tibia and values above 90° indicate dorsiflexion; see Bolsterlee et al. (2017) for details). The dorsiflexed position was the maximal dorsiflexion angle the participant could comfortably achieve (108 ± 7^∘^). Thus, the ankle angles differed slightly between participants (Table 1). The change in muscle-tendon length between the plantarflexed and dorsiflexed position was estimated using regression equations (Grieve, Pheasant & Cavanagh, 1978).

All scans were made using a 3T MRI scanner (Achieva 1.2, Philips Medical Systems, Best, The Netherlands) with a 16-element SENSE XL torso coil. In each joint position, 75 transverse anatomical images and DTI images were obtained covering the entire cross-section of the left lower leg from the proximal end of the tibia to the ankle (or for taller subjects, close to the ankle). The settings for the anatomical scan were: TSE sequence, TR/TE 1842/8 ms, field of view (FOV) 180 mm, acquisition matrix 288 × 215 (reconstructed to 960 × 960), voxel size 0.1875 × 0.1875  × 5 mm and scan time 320 s. The settings for the DTI scans were: DT-EPI sequence, TR/TE 8522/63 ms, FOV 180 mm, voxel size 1.875 × 1.875 × 5 mm, 16 gradient directions on a hemisphere, number of signal averages 2, *b* = 500 s/mm2 (B0 image with *b* = 0 s/mm2) and scan time 298 s.

The DTI data were corrected for eddy current distortions (Andersson & Sotiropoulos, 2016) and filtered with a local principal component analysis filter that smooths the raw DTI data while maintaining sharp transitions between adjacent muscles (Manjon et al., 2013). Subsequently, the diffusion tensor was reconstructed using DSI Studio (dsi-studio.labsolver.org; Yeh et al., 2013), from which the primary, secondary and tertiary diffusion eigenvalues and eigenvectors were derived. A map of the primary eigenvector (which indicates the primary diffusion direction, and thus the fibre orientation) was created.

### Muscle segmentation

**Figure 1:**
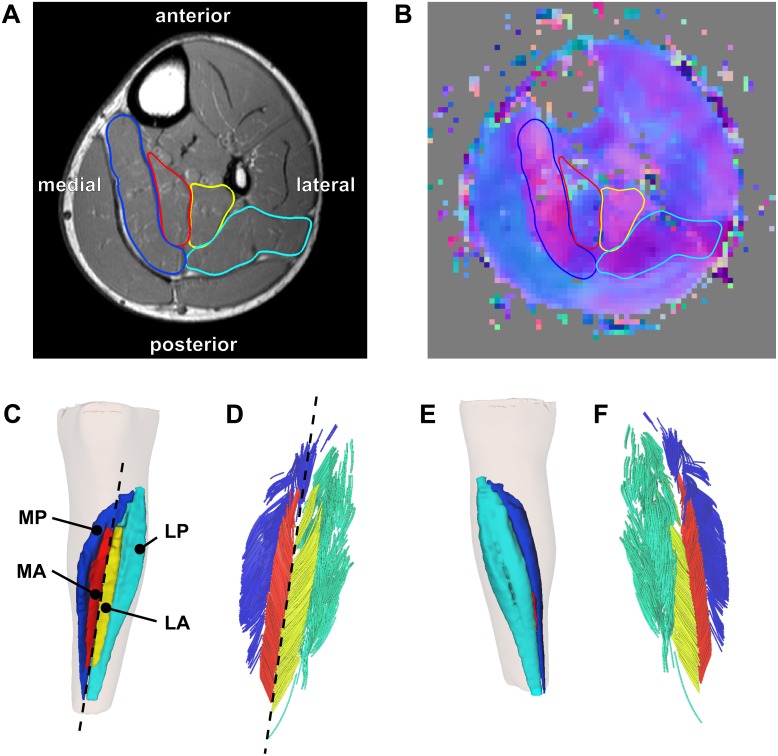
Reconstruction of the architecture of the human soleus muscle using MRI and DTI. (A) Transverse T1-weighted MRI slice approximately midway between the ankle and the knee. The four compartments of the soleus are outlined (blue, medial-posterior (MP); cyan, lateral-posterior (LP); red, medial-anterior (MA); yellow, lateral-anterior (LA)). (B) Corresponding slice of the primary eigenvector map from the DTI data. Different colours indicate different diffusion directions (i.e., muscle fibre directions). The outlines of the soleus compartments from the anatomical scan are overlayed to show that the four compartments have different fibre orientations. (C) Anterior view of the left lower leg (top is superior) showing the 3D reconstruction of the surface of all muscle compartments based on the outlines on the anatomical scan. The skin is shown as a transparent overlay. (D) Anterior view of 3D fascicle reconstructions (after extrapolations of fibre tract end points to the muscle surface) of the same muscle as in (C). Fascicles are coloured per compartment. The dashed line in (C) and (D) represents the long axis of the muscle relative to which pennation angles were calculated. (E) Posterior view of the left lower leg showing the posterior compartments of the soleus and the skin (transparent). (F) Posterior view of 3D fascicle reconstructions.

The soleus muscle was manually outlined on all of the slices of the T1-weighted anatomical scans that the muscle was visible on (on average, 59 slices per scan with a spacing of 5 mm between slices) using 3D Slicer (http://www.slicer.org—Fedorov et al., 2012). All segmentations were performed or checked by a researcher (author TF) experienced in studying soleus anatomy (Finni et al., 2003a; Finni et al., 2003b; Hodgson et al., 2006). It was apparent that the muscle was subdivided in four compartments (Fig. 1A): medial-anterior (MA), lateral-anterior (LA), medial-posterior (MP) and lateral-posterior (LP), which is consistent with previous anatomical descriptions (Agur et al., 2003; Finni et al., 2003a).

On most slices of the anatomical scan the boundaries of each compartment could clearly be identified, but in the more proximal regions the boundaries within the muscle were sometimes unclear. To guide and check the manual outlining, the segmentations made on the anatomical scans were overlayed on the primary eigenvector map derived from the DTI data using image processing software ITK-SNAP (http://www.itksnap.org; Yushkevich et al., 2006). The primary eigenvector map contains a 3-component direction vector indicating the 3D fibre direction in each voxel of the DTI scan, which can be visualised as a colour-coded map where different colours indicate different fibre orientations (Fig. 1B). As different muscle compartments have clearly different fibre orientations, the map assisted in drawing the boundaries between compartments on slices where the boundaries were unclear on the anatomical scan. For this procedure we used ITK-SNAP to overlay the primary eigenvector map on the anatomical scan with interactively changeable transparency, facilitating optimal use of both anatomical and DTI data sources for manual segmentation. As an indicator of reliability of the segmentation, we calculated root mean squared differences in muscle volume between scans obtained at short and long muscle lengths. As muscle volume is likely to change less than 2% with passive lengthening (Bolsterlee et al., 2017), larger differences in volume between muscle lengths presumably indicate errors in segmentation.

### Muscle architecture measurements

From the segmentations, 3D triangulated surface models were created for all compartments using the MATLAB-based iso2mesh toolbox (Figs. 1C and 1E; Fang & Boas, 2009). Muscle volume of each compartment was calculated as the volume of the surface models. Muscle length was calculated as the distance between the most proximal and the most distal point of the surface models of all compartments combined, projected along the long axis of the muscle. The long axis of the muscle was approximated by a line connecting a proximal and distal point on the anterior surface of the soleus between the medial-anterior and lateral-anterior compartment (Figs. 1C–1D).

Deterministic DTI fibre tracking algorithms built into DSI Studio (Yeh et al., 2013) were used to generate 1,000 fibres in each muscle compartment. Fibre tracking is the procedure of generating curves that, starting from a seed point, follow the primary direction of diffusion bi-directionally through a DTI scan volume (Mori et al., 1999). When performed in muscle tissue, these curves follow the fibre orientation throughout a muscle (Damon et al., 2002) and, when appropriate stopping criteria are defined (Heemskerk et al., 2009), they resemble muscle fibres (Damon et al., 2017). We used randomly placed seed points located inside the muscle but at least two voxels away from the muscle surface. Fibre tracking is often inaccurate at the boundary of the muscle (Sinha et al., 2015), possibly because of erroneous estimation of the fibre direction in voxels that contain signal from more than one muscle (Oudeman et al., 2016). Therefore, we terminated fibre tracking when the tract entered the boundary layer of a muscle. The seed and boundary regions were created by resampling the masks created on the anatomical scans to the resolution of the DTI scans. We used fibre tracking settings similar to those used previously by our group and others : 0.1 ≤ fractional anisotropy ≤ 0.5, 1⋅10^−3^ mm^2^/s ≤ mean diffusivity ≤2⋅10^−3^ mm^2^/s, maximum angle between subsequent tract segments = 10° , 15 mm ≤ fibre tract length ≤ 200 mm, step size = 1 mm. Seeding was continued until 1,000 tracts satisfying these constraints were found.

Fibre tracts frequently do not terminate on aponeuroses or tendons, and thus the ends of fibre tracts do not represent the ends of muscle fascicles. To find origin and insertion points for each fibre tract we used algorithms to ensure that fibre tracts attach to aponeuroses (or, more precisely, to 3D surface models generated from the manually outlined muscles) (Bolsterlee et al., 2017). Briefly, the 3D surface models from the anatomical scans were overlayed on the fibre tracts and a third-order polynomial curve was fitted in 3D to the fibre tracts. The polynomial curve was linearly extended at both end points using the slope of the curve at the end point until the extension intersected the muscle surface. The polynomial curves fitted on the raw fibre tracts, including their extrapolations to the muscle surface, will be referred to as fascicles. The extrapolation procedure reduces the variability of fascicle lengths within a muscle and presumably makes the results less sensitive to the fibre tracking settings than if tracts are not extrapolated (Bolsterlee et al., 2017).

Fascicle lengths were calculated as the sum of the length of the polynomial curves (fitted on the raw fibre tracts) and the extrapolations. We also calculated fascicle lengths as a fraction of whole-muscle length. Pennation angles were calculated in 3D as the angle between the line connecting the origin and insertion of a fascicle and the long axis of the muscle (Figs. 1C–1D). Using the Frenet–Serret formula, the curvature of a fascicle (expressed as 1/radius (m^−1^)) was calculated as the mean curvature of 100 equidistant points along the polynomial curve of that fascicle.

Of the 1,000 fascicles that were reconstructed in each compartment, only fascicles extended by less than 50% of their total length and less than 20 mm were included in further analyses. All fascicles were visually inspected for plausibility and to verify the distribution of fascicles throughout each compartment (Figs. 1D and 1F). Mean muscle architecture measurements for a compartment were calculated by averaging architecture measurements from all successfully reconstructed fascicles in that compartment. Within each compartment, physiological cross-sectional area (PCSA) was calculated by dividing muscle volume by fascicle length.

### Statistics

We used linear mixed models to investigate differences between compartments in architecture (mean fascicle length, pennation angle and curvature) at the short muscle length. In these models, subjects were assigned random intercepts and there were fixed effects for muscle compartment.

We also used linear mixed models to quantify changes in architecture with passive lengthening. These models estimated the change in architecture per centimetre change in muscle-length, and tested for differences between compartments in change in architecture with passive lengthening. Subjects were assigned random intercepts and there were fixed effects for muscle compartment, muscle length and the interaction between muscle compartment and muscle length.

### Cadaver measurements

**Figure 2:**
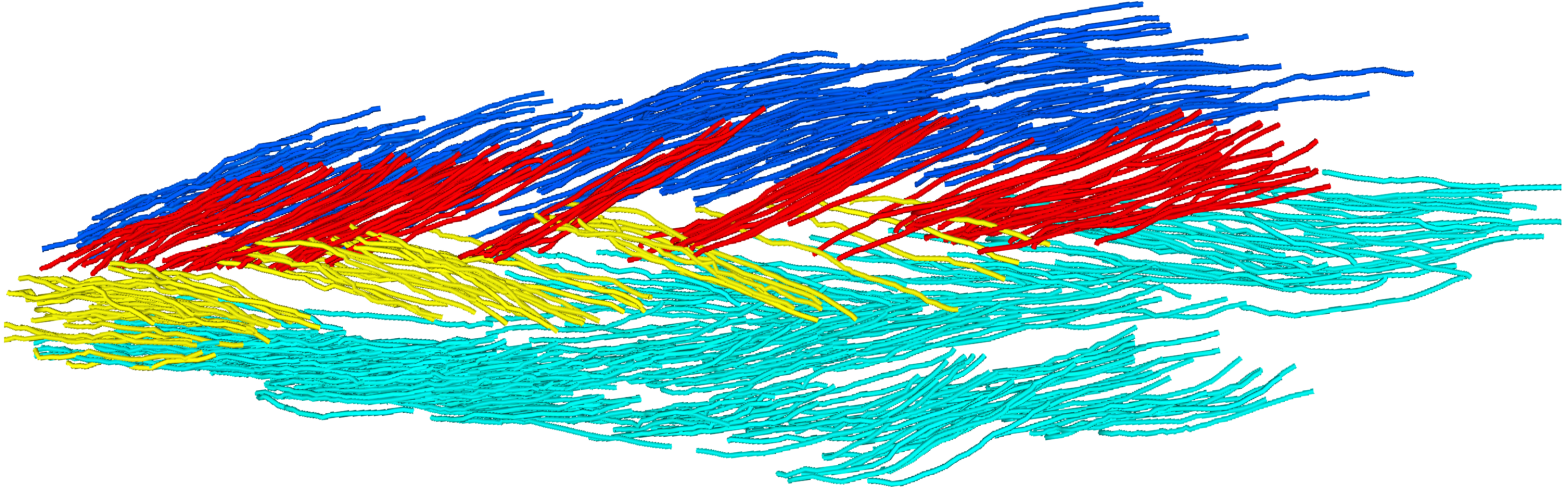
Anterior view of a 3D fascicle reconstruction of a cadaveric soleus muscle. The reconstruction was made through dissection and 3D digitisation. Note the resemblance with the *in vivo* reconstruction from DTI in Fig. 1. To make the reconstruction, the tip of the digitiser was run along the course of a fascicle while recording the 3D coordinates of the tip. After digitisation of a fascicle, the fascicle (or a bundle of fascicles) was carefully removed. This process was repeated until all fascicles were removed. Colours indicate different compartments (same colours as in Fig. 1).

To confirm the soleus anatomy reconstructed with DTI, we measured the architecture of two cadaveric human soleus muscles using 3D microdissection techniques similar to the techniques used by Lee et al. (2015). The muscles were harvested from the right legs of fresh-frozen cadavers of a 67-year old male (muscle 1; muscle length: 36.5 cm) and a 90-year old male (muscle 2; muscle length: 32 cm). After gross dissection, the soleus muscles were fixed by submerging the tissue in a 10% neutral buffered formalin solution for 11 weeks. The architecture of the fixed soleus was reconstructed using a 3D spatial digitiser (MicroScribe G2X; Immersion Corp., San Jose, CA, USA), connected to a laptop computer. The tip of the digitiser was run from origin to insertion of a clearly visible fascicle bundle while recording the 3D coordinates of the tip with a sampling frequency of 20 Hz (leading to ∼1 mm spacing between points; Fig. 2). After digitisation, the fascicle bundle was removed carefully using scalpel and tweezers, exposing deeper fascicles which were then digitised and removed. This procedure was repeated until no muscle tissue was left. We digitised only those fascicles whose entire course from origin to insertion could clearly be identified. To limit movement of the muscle relative to the base of the digitiser (i.e., relative to the fixed coordinate system of the digitiser), the muscle was cast in a custom-made silicon mould during dissection and digitisation.

Fascicle length was calculated as the sum of the lengths of the line segments connecting the points along the course of one fascicle. Pennation angle was calculated in 3D as the angle between the line connecting the first and last point on a fascicle, and the line connecting two points on the anterior surface of the medial septum (considered an approximation of the line of action of the soleus). Mean fascicle lengths and pennation angles were calculated for all four compartments of both muscles.

We did not control for ankle angle during gross dissection of the soleus muscle, and muscle length was not fixed during fixation, so we do not know the ankle angle corresponding to the architecture measurements reported for the dissections. During fixation the muscles were hung vertically from their proximal ends in a cylindrical tube with a small weight (∼0.5 kg) attached to the distal end, so the tension in the tissue was small. Therefore, the muscle length is likely to correspond to a more plantarflexed ankle position, which is comparable to the ‘short’ condition in the *in vivo* data.

## Results

**Figure 3:**
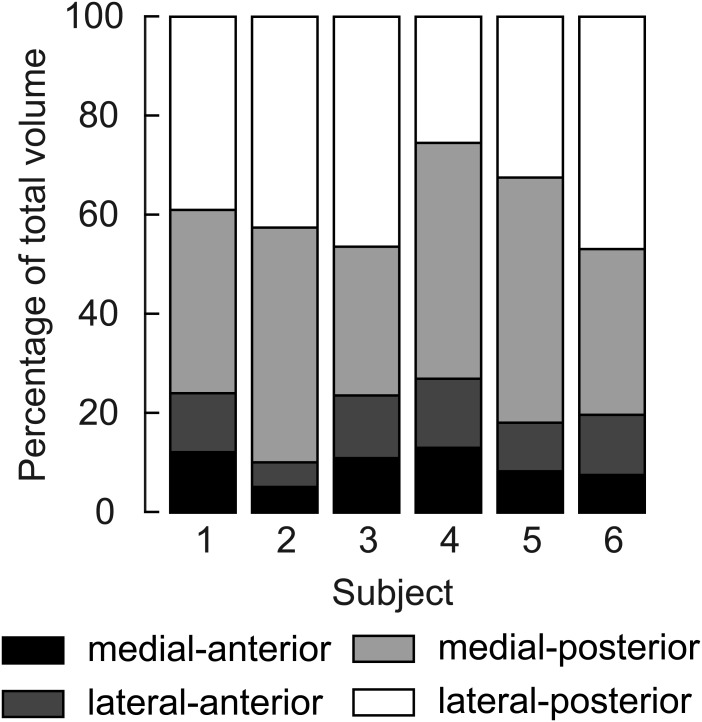
Relative volume of the four compartments of the human soleus. In all subjects the posterior compartments were substantially larger than the anterior compartments, but there was considerable inter-individual variation. The volume of a compartment is the mean of the volumes measured at the short and the long length.

Four distinct compartments were apparent on the MRI and DTI scans of all six participants (Fig. 1A). The posterior compartment had, on average, four times the volume of the anterior part, although there was some inter-individual variation (Fig. 3, Table 2). The average total muscle volume was 356 ± 58 cm^3^. On average, muscle volumes in all compartments were similar between measurements made at short and long muscle lengths (Table 2). The root mean squared difference between compartment volumes measured at short and long muscle lengths was 5.7 cm^3^ or 6.3% of the average compartment volume. The root mean squared difference between total muscle volumes (sum of four compartments) measured at short and long lengths was 14.1 cm^3^ or 3.9% of the average muscle volume.

**Table 2:** Muscle architecture per soleus compartment at short muscle lengths, long muscle lengths and the change in architecture from short to long lengths (change = long-short). Values are means ± SDs over all subjects of the average parameter value of all fascicles in a muscle compartment. For the whole muscle (last three rows), values are means ± SDs over all subjects of the summed (for volume and PCSA) or average (for fascicle length, pennation and curvature) value over all compartments.

Compartment	Condition	Volume (cm^3^)	PCSA (cm^2^)	Fascicle length (mm)	Pennation angle (°)	Curvature (/m)
Medial-anterior	Short	33.5 ± 10.1	9.3 ± 3.5	37.7 ± 9.3	22.0 ± 4.7	15.3 ± 4.4
	Long	33.5 ± 11.0	6.7 ± 2.4	51.9 ± 13.3	17.3 ± 3.1	7.9 ± 2.0
	Change	0.0 ± 2.1	−2.6 ± 1.8	14.2 ± 7.3	−4.7 ± 2.5	−7.4 ± 3.8
Lateral-anterior	Short	40.4 ± 14.4	10.9 ± 4.1	37.7 ± 10.4	27.1 ± 4.1	16.1 ± 3.9
	Long	37.0 ± 13.2	8.0 ± 2.3	45.9 ± 9.2	18.6 ± 1.8	7.7 ± 1.2
	Change	−3.4 ± 4.7	−3.0 ± 2.8	8.3 ± 3.7	−8.5 ± 2.6	−8.4 ± 3.1
Medial-posterior	Short	146.6 ± 29.1	41.5 ± 8.8	36.0 ± 7.5	38.3 ± 7.7	19.7 ± 4.3
	Long	139.4 ± 25.1	30.7 ± 5.1	45.9 ± 7.5	24.5 ± 3.7	11.4 ± 2.5
	Change	−7.2 ± 5.5	−10.9 ± 4.2	9.9 ± 1.3	−13.9 ± 5.3	−8.3 ± 3.6
Lateral-posterior	Short	141.7 ± 47.4	40.1 ± 12.5	35.7 ± 7.9	34.9 ± 4.3	18.6 ± 3.2
	Long	139.2 ± 50.1	29.9 ± 7.8	46.2 ± 10.1	24.2 ± 2.4	10.8 ± 2.8
	Change	−2.5 ± 5.4	−10.2 ± 5.6	10.5 ± 3.7	−10.7 ± 4.0	−7.8 ± 2.4
Whole-muscle	Short	362.2 ± 59.2	101.8 ± 19.1	36.8 ± 8.4	30.6 ± 3.3	17.4 (3.6)
	Long	349.1 ± 57.6	75.2 ± 8.0	47.5 ± 8.6	21.1 ± 1.9	9.4 ± 1.9
	Change	−13.1 ± 9.7	−26.6 ± 11.5	10.7 ± 2.7	−9.4 ± 2.6	−8.0 ± 2.9

From the 1,000 fibres tracked in each muscle compartment, on average, 600 fascicles were successfully reconstructed (range across muscle compartments 261–907). These fascicles were extrapolated by 11.7 ± 1.3 mm (29.4 ± 4.5% of the fascicle length). In all compartments fascicles were reconstructed throughout most of the muscle belly (Figs. 1C–1F), although fewer fascicles were successfully reconstructed in the most proximal and distal ends of the soleus. The DTI-based muscle fascicle reconstructions confirmed the presence of a bipennate anterior compartment and a unipennate posterior compartment in all subjects (Fig. 1).

**Figure 4:**
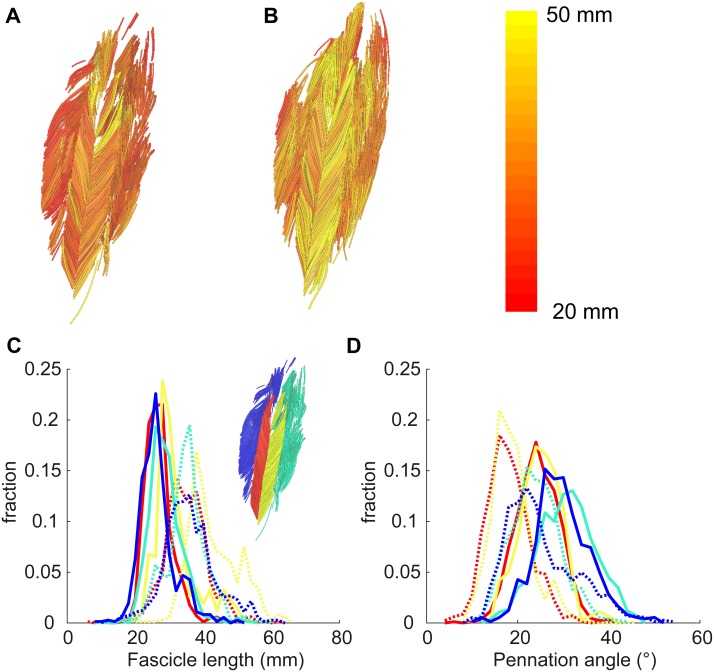
Example of 3D reconstruction and muscle architecture measurements. Anterior view of the soleus muscle of one subject with the muscle at a short (A) and a long length (B). Fascicles are coloured according to their length. Distribution of (C) fascicle lengths (bin size = 2 mm) and (D) pennation angle (bin size = 2°) for all four compartments (same colours as in Fig. 1; see also inset in C). The solid and dashed lines present measurements obtained at the short and long muscle length, respectively. Fraction, fraction of total number of successfully reconstructed fascicles.

**Figure 5:**
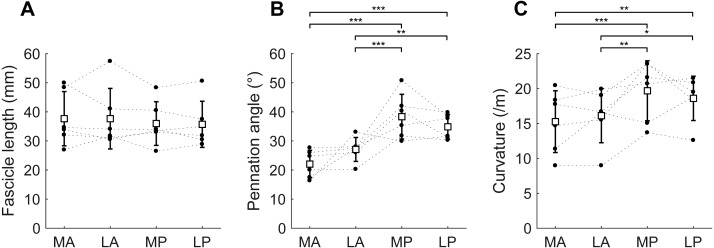
Muscle architecture of the soleus at the short muscle length. (A) Fascicle length, (B) pennation angle and (C) curvature, grouped by muscle compartment. White squares and vertical bars indicate mean value ± 1 SD over all subjects. Thin dashed lines connect the measurements made on one subject. Horizontal bars indicate statistically significant differences between compartments (^∗^*p* < 0.05, ^∗∗^*p* < 0.01, ^∗∗∗^*p* < 0.001). MA, medial-anterior; LA, lateral-anterior; MP, medial-posterior; LP, lateral-posterior.

At the short muscle length, fascicle lengths, pennation angles and curvatures were 36.8 ± 8.4 mm, 30.6 ± 3.3° and 17.4 ± 3.6 /m (mean ± SD across subjects of the mean value of all four compartments), respectively (Fig. 4, Table 2). Average fascicle lengths in the soleus ranged from 29 to 51 mm between individuals (Fig. 5). As a proportion of muscle length, average fascicle lengths were 0.13 ± 0.03. Within individuals we did not find statistically significant differences between compartments in fascicle lengths (Fig. 5A). Pennation angles, however, were significantly larger in the posterior compartments than in the anterior compartments by, on average, 12° (Fig. 5B). Also, curvatures were 4/m larger in the posterior compartments compared to the anterior compartments (Fig. 5C). We did not detect statistically significant differences in architecture between lateral and medial compartments.

**Figure 6:**
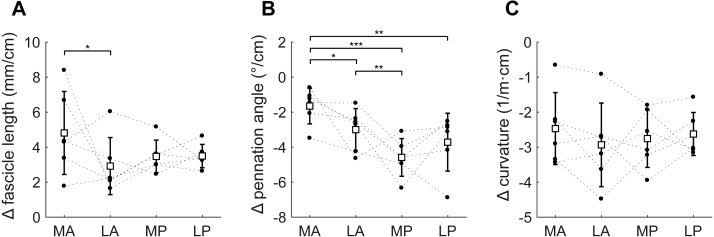
Change in architecture of the soleus with passive muscle lengthening. (A) Fascicle length, (B) pennation angle and (C) curvature, grouped by muscle compartment. Values are parameter change per centimetre increase in muscle-tendon length. White squares and vertical bars indicate mean value ± 1 SD over all subjects. Thin dashed lines connect the measurements made on one subject. Horizontal bars indicate statistically significant differences between compartments (* *p* < 0.05, ** *p* < 0.01, *** *p* < 0.001). MA, medial-anterior; LA, lateral-anterior; MP, medial-posterior; LP, lateral-posterior.

Passive lengthening of the soleus muscle-tendon unit by 30 ± 8 mm resulted in changes in architecture that were consistent between subjects (Fig. 6). For every centimetre that the muscle-tendon unit lengthened, fascicle lengths increased by 3.7 ± 0.8 mm, pennation angles decreased by 3.2 ± 0.9° and curvatures decreased by 2.7 ± 0.8 /m. Although the fascicle length change was significantly different between the medial-anterior and lateral-anterior compartment, we generally found small and non-significant differences between medial and lateral compartments in changes in architecture. Pennation angles decreased more in posterior compartments (−4.2 ± 1.1°/cm muscle-tendon lengthening) than in anterior compartments (−2.3 ± 0.8°/cm; Fig. 6B).

Cadaver dissections confirmed the four-compartment architecture of the soleus in both cadaveric muscles. Lengths and pennation angles of 994 fascicles were measured in two muscles with a minimum of 58 fascicles per compartment (Table 3). Dissection measurements of fascicle lengths were similar to those measured using DTI. Dissection measurements of anterior compartment pennation angles were similar to those measured with DTI, but dissection measurements of posterior compartment pennation angles were smaller than those measured by DTI. In contrast to measurements made with DTI, dissection measurements of pennation in the posterior and anterior compartments were similar.

**Table 3:** Muscle architecture per soleus compartment of two human cadaveric soleus muscles measured by microdissection techniques. The values represent the mean ± SD of all fascicles that were digitised in that compartment.

	Compartment	*n*	Fascicle length (mm)	Pennation angle (°)
Muscle 1	Medial-anterior	102	37.9 ± 4.9	25.0 ± 5.9
	Lateral-anterior	97	34.3 ± 5.0	28.8 ± 10.3
	Medial-posterior	123	31.4 ± 5.1	19.6 ± 4.7
	Lateral-posterior	68	27.9 ± 7.1	24.2 ± 7.4
Muscle 2	Medial-anterior	90	50.9 ± 4.8	19.9 ± 4.6
	Lateral-anterior	58	46.2 ± 5.0	18.0 ± 4.4
	Medial-posterior	162	43.9 ± 3.1	16.8 ± 3.1
	Lateral-posterior	294	42.0 ± 4.8	15.0 ± 3.9

## Discussion

This study used novel DTI-based techniques to discern the complex 3D anatomy of the human soleus muscle *in vivo*. The data confirmed the four-compartment structure of the soleus previously described in cadaver muscles. There was evidence of differences in pennation angles and curvature, but not fascicle lengths, between the posterior and anterior compartments. Importantly, this is the first study to report inter-compartment differences in change in architecture of the human soleus with passive lengthening.

The gross anatomical data we present here are largely comparable with previous studies with four identified compartments (Agur et al., 2003; Hodgson et al., 2006). However, we did not observe, with either the imaging procedures or the dissection, a fifth marginal soleus compartment, as described by Agur et al. (2003). The marginal soleus was also not discerned in the Visible Human Dataset (Sinha et al., 2011).

Fascicle lengths and pennation angles fall within the range previously reported on cadaver muscles (Agur et al., 2003; Ward et al., 2009; Spoor et al., 1991) and *in vivo* using ultrasound (Stenroth et al., 2016; Kawakami, Ichinose & Fukunaga, 1998; Maganaris, 2001; Maganaris, Baltzopoulos & Sargeant, 1998). Although fascicle lengths between compartments were similar, there was considerable variation in average fascicle lengths between subjects, ranging from 29 to 51 mm at the short muscle length and 37 to 61 mm at the long length. Soleus muscle volumes in our dataset (mean 356 cm^3^) were larger than those measured in cadaver muscles (260 cm^3^; Ward et al., 2009), but smaller than previously measured *in vivo* (489–550 cm^3^; Hodgson et al. (2006), Fukunaga et al., 1992). Sarcopenia in the muscles of the elderly cohort studied by Ward et al. likely explains the smaller soleus volumes in their sample. The predominantly male participants (11 male, one female) studied by Fukunaga et al. were, on average, 5% taller and 17% heavier than our participants (three male, three female), explaining at least partially why the soleus in Fukunaga’s sample was larger. Hodgson et al. (2006) did not report the gender, height or weight of the three participants for which they reported muscle volumes. In our six participants, the posterior compartments were 2.7 to 8.9 times larger than the anterior compartments, confirming previous reports of large inter-individual variations in relative volume between compartments (Hodgson et al., 2006). The larger PCSA and smaller pennation angles of the posterior compartments indicate a muscle design more geared towards high force production. The smaller PCSA and pennation angles of the anterior compartment may indicate a role more geared towards establishing muscle excursion, rather than force production. However, we did not detect differences between compartments in fascicle length/muscle length ratio, which indicates whether the design of a muscle is more geared towards providing large excursions (high values) or high forces (low values). The low ratio (0.13 ± 0.3) is in line with previous cadaver measurements (0.11; Ward et al., 2009) and confirms the role of the soleus in generating high forces over a small range of lengths.

This study is the first to report inter-compartment differences in change in architecture of the human soleus with passive lengthening. The medial anterior compartment showed the most pronounced change in length (∼14 mm) that was significantly different from that in the lateral anterior compartment. We observed larger fascicle rotations in the posterior compartments, which could be indicative of a functionally different role of the posterior and anterior compartments. Functional partitioning of the soleus was suggested when it was found that nerve branching in the human soleus follows the structural compartmentalisation of the muscle (Loh, Agur & McKee, 2003). This provides a possible mechanism for independent control of different compartments. A new observation contributed by the current study is that this compartmentalisation is also reflected in differences in passive mechanical properties of the compartments. To the extent that the observed inter-compartmental differences are functionally important (English, Wolf & Segal, 1993), it may be difficult to infer the function of the whole soleus from measurements made on just one compartment, as is commonly done in ultrasound studies.

Few other studies have reported changes in 3D muscle architecture in the soleus with passive lengthening. Sinha et al. (2011) also used DTI techniques and reported changes in fascicle orientation (or, more precisely, changes in the primary eigenvector of the diffusion tensor) up to 46° with 30° ankle rotation—much larger than the changes we report here (9.4° change in pennation over 39° ankle rotation). Whereas Sinha and colleagues reported changes in the primary eigenvector relative to the long axis of the scanner, we measure pennation angles from fascicles reconstructions relative to the long axis of the muscle. The fascicle reconstructions were bound by anatomical constraints, and implausible fascicles were excluded, presumably making our measurement more accurate. Rana, Hamarneh & Wakeling (2013) used 3D ultrasound techniques to measure changes in fibre orientation (but not fascicle lengths) with plantarflexion contractions and ankle rotation. They reported only 0.8° rotation of fascicles in the soleus (relative to the muscle’s long axis) with 45° ankle rotation, which is surprisingly small compared to fibre rotation measured over the same ankle joint range using 2D ultrasound (15°; Maganaris, 2001). The change in pennation angles and fascicle lengths we report here are much closer to 2D ultrasound-measured values from the posterior compartment (Maganaris, 2001), and physiologically more plausible. Note that the values we report represent a linear (i.e., average) estimate of the possibly non-linear effect of muscle-tendon lengthening on changes in muscle architecture (Herbert et al., 2011).

The DTI-based reconstruction techniques used here improve on conventional DTI fibre tracking algorithms. Tractography algorithms generate curves that follow the direction in which most diffusion occurs (i.e., the muscle fibre direction) throughout a muscle. The lengths of these curves are often interpreted as muscle fibre lengths, and the orientation of the curves is often used to calculate pennation angles (Sinha et al., 2011; Oudeman et al., 2016). Unfortunately, the fibre tracts are only moderately reproducible (Heemskerk et al., 2009). Application of this approach to measuring fascicle lengths in the soleus has resulted in measurement of fibre lengths ranging from ∼1–2 cm (Sinha et al., 2011) to 5–6 cm (Oudeman et al., 2016). These values depart substantially from the findings of the most detailed dissection study, which reported fascicle lengths of 3–4 cm (Agur et al., 2003), and other cadaver studies that report similar measurements (Ward et al., 2009; Spoor et al., 1991).

In the present study we used information from anatomical scans about the location of the aponeuroses to force fibre tracts to terminate on tendons or aponeuroses. In our opinion, these constraints are necessary to obtain realistic measurements of muscle architecture. We also expect that constraining fibre tracts to end on aponeuroses reduces the sensitivity of the architecture measurements to variations in fibre tracking settings; however, this hypothesis requires formal testing. Although direct validation was not possible, the consistent and physiologically plausible changes in architecture we observed in all muscle compartments in all participants provide some evidence for the validity of our techniques. Extrapolation of fibre tracts to aponeuroses requires segmentation of muscles from scans, which is time-consuming and prone to error. We found that manual segmentation was aided greatly by overlaying the primary eigenvector map on the anatomical scan (as detailed in the Methods section) to make optimal use of information about muscle boundaries from the two data sources. The root mean squared difference in volumes of the soleus’ compartments between short and long lengths was 6.3%, indicating a reasonable reliability of manual segmentation in this complex muscle. Some, but probably not all, of this difference may be attributed to actual changes in volume with passive stretching, as we showed earlier in this dataset that medial gastrocnemius muscle volume reduced by 1.6% from short to long muscle lengths (Bolsterlee et al., 2017). We did not determine the extent to which inaccuracies in manual muscle segmentations have propagated to errors in muscle architecture measurements. Future development of (semi-)automatic muscle segmentation algorithms will be of great benefit to obtain accurate, reproducible measurements of muscle architecture from DTI. A possible pathway to development of such algorithms is to use information from both anatomical and DTI scans, rather than only from anatomical scans (e.g., Gilles & Magnenat-Thalmann, 2010).

The DTI-based measurements of muscle architecture were compared to measurements made using microdissection techniques on two cadaveric muscles. Similar measurements of fascicle lengths and pennation angles in the anterior compartments were obtained with the two approaches. However, the dissection did not reproduce the finding made with DTI that pennation angles in the posterior compartments are larger than in the anterior compartments. This inconsistency between the dissection and DTI data might be explained by an altered orientation of the posterior compartments relative to the long axis of the muscle caused by removing the muscle from the surrounding anatomical structures (e.g., the gastrocnemius). It is also possible that age-related changes affect the pennation angles of the posterior compartments differently compared to the anterior compartments.

Unfortunately, we were not able to obtain high-quality DTI measurements from the muscles that we dissected. Prior to dissection we scanned the cadaver muscles using the same DTI scanning protocol as used *in vivo*, and we applied the same algorithms for reconstruction, but the quality of the reconstructions was poor. It is likely that freeze-thawing or fixing the muscle altered the diffusion properties of the tissue. It is difficult or impossible to procure whole human muscles that have not been freeze-thawed or fixed. This precludes direct validation of DTI measurements of muscle architecture on human muscles. For now, direct validation of DTI-based measurement of muscle architecture are best carried out by comparing DTI and dissection measures on animal muscles (Schenk et al., 2013; Damon et al., 2002), or with phantoms that resemble muscle tissue (Berry et al., 2017).

Classical anatomical textbooks provide qualitative descriptions and schematic images of muscle architecture which are valuable for gaining a general understanding of musculoskeletal function. But more detailed quantitative analyses, for instance with muscle models, require quantitative data on muscle architecture. All of the existing datasets of muscle architecture have been obtained from cadaver muscles, often obtained from the cadavers of elderly people at least some of whom have sarcopenia. The techniques we present here will be useful in the development of three-dimensional, quantitative atlases of human muscle architecture *in vivo*.

## Conclusions

Using a novel DTI-based method this study presented comprehensive data of the 3D architecture of the four compartments of human soleus muscle *in vivo*. Importantly, the results provided a first look on 3D changes in the compartmentalised soleus muscle during passive lengthening that may provide reference values for future muscle models. The DTI techniques that we applied here to quantify the architecture of the soleus could be used to develop comprehensive, quantitative atlases of human muscle architecture.

##  Supplemental Information

10.7717/peerj.4610/supp-1 Data S1 Cadaver data: architectural measurements through 3D dissection and digitisation

## Additional Information and Declarations

### Data Availability

The following information was supplied regarding data availability: Both the *in vivo* and the cadaver data are made available through our institutional repository. The raw data including a data description can be downloaded from:
https://researchdata.neura.edu.au/2018/01/08/01 (*in vivo* data: MRI and DTI scans and architectural measurements) and https://researchdata.neura.edu.au/2018/01/08/02 (cadaver data: architectural measurements through 3D dissection and digitisation).
